# Hematologic tests and their association with the severity of COVID-19 and periodontitis in hospitalized patients: a case–control study

**DOI:** 10.1186/s12903-023-03208-3

**Published:** 2023-07-11

**Authors:** Janet Moradi Haghgoo, Parviz Torkzaban, Maryam Farhadian, Nazli Rabienejad, Sayed Ali Moosavi Sedeh

**Affiliations:** 1 Department of Periodontics, School of Dentistry, Hamadan University of Medical Sciences, Hamadan, Iran; 2 Dental Research Center, Hamadan University of Medical Sciences, Hamadan, Iran; 3 Department of Biostatistics, School of Public Health and Research Center for Health Sciences, Hamadan University of Medical Sciences, Hamadan, Iran

**Keywords:** Periodontitis, COVID-19, Severity, Blood tests, Hematological tests, Hematology

## Abstract

**Background:**

The presence of comorbidities, especially those with a chronic inflammatory nature such as periodontitis, can facilitate COVID-19 progression toward more severe forms. Both of these diseases can affect systemic health and alter hematological test results. In this study, we decided to investigate COVID-19 and periodontitis’ possible interaction with these alterations.

**Methods:**

Hospitalized patients with a definitive diagnosis of COVID-19 were included. Controls had mild to moderate COVID-19, while cases had severe to critical COVID-19. Periodontal examination was done for each patient. Relevant medical and hematological data were extracted from patient’s hospital files.

**Results:**

A total of 122 patients entered the final analysis. The minimum white blood cell counts were associated with the severity of periodontitis. The interaction between periodontitis and COVID-19 was associated with increased minimum white blood cell counts and decreased platelet counts. COVID-19 severity was associated with increased venous oxygen saturation, prothrombin time, the maximum partial thromboplastin time, the maximum and average urea, the maximum creatinine, the maximum potassium, and lactate dehydrogenase, and decreased sodium levels.

**Conclusions:**

Results of this study showed that several blood parameters were associated with periodontitis, COVID-19, or the interaction between them.

## Background

COVID-19 has so far caused over 768 million confirmed cases of infection and over 6.9 million deaths [1]. While the disease course is mild in the majority (81%) of cases, certain factors can facilitate the disease’s progression towards more severe forms [2]. The presence of comorbidities, especially those with a chronic inflammatory nature, such as diabetes, is such a factor [3]. Among the comorbidities that may have an effect on COVID-19 severity is periodontitis.

Periodontitis is characterized by the progressive destruction of the supporting structures of the tooth (i.e., periodontium), eventually leading to tooth loss [4]. Having a chronic inflammatory nature, it has been documented that periodontitis has significant effects on systemic health, with a prominent example being its interaction with diabetes. Diabetic patients will experience worse glycemic control with untreated periodontitis, and proper management of periodontitis helps with glycemic control in diabetic patients [5].

With both COVID-19 and periodontitis causing deleterious effects beyond the organs they involve, hematological tests may show changes associated with these effects. Changes in complete blood count parameters, liver enzymes, coagulation tests, biomarkers such as C-reactive protein, and other tests have been reported for either or both diseases [6–12].

The possible interactions between COVID-19 and periodontitis include: presence of entry receptors in the periodontium [13, 14], the presence of SARS-CoV-2 in the gingival sulcus [15], their effects on comorbidities and systemic health, and the possible interactions on hematological tests. Furthermore, recent research has revealed a possible association between COVID-19 and periodontitis [16–18]. Based on the points discussed before, we decided to investigate the association between COVID-19, periodontitis, and hematological tests. To objectively assess COVID-19 severity, evaluate more severe forms, and perform hematological tests in a controlled environment, we decided to conduct this study in a hospital setting on hospitalized patients.

## Methods

### Study design

This study was designed in a case‒control format. All patients had a confirmed COVID-19 diagnosis and were assigned to either case or control groups based on their COVID-19 severity. Patients with mild to moderate COVID-19 were considered controls, while patients with severe to critical COVID-19 were considered the case group. All patients were periodontally examined, had their laboratory test data extracted, and were followed up for any change in their COVID-19 severity.

In the first part of the study, we evaluated the distribution of periodontal disease severity among the case and control groups to evaluate any possible association between periodontitis and COVID-19 (i.e., the primary exposure variable was periodontitis severity, while the primary outcome variable was COVID-19 severity). Then we evaluated the levels of hematological parameters across periodontitis and COVID-19 severity groups, to investigate any possible association between periodontitis, COVID-19, and hematological tests. Thus, the primary objective of this study was to investigate the association between COVID-19, periodontitis, and the results of hematological tests. The investigation into associations between COVID-19, periodontitis, and related clinical parameters were performed in the other part of this study. This study conforms to the STROBE guidelines for reporting its results.

### Setting

Patients were enrolled in the study at a hospital with dedicated COVID-19 wards and intensive care units (ICUs). A single researcher was responsible for patient enrollment at each hospital visit. Hospital visits were scheduled randomly with a median interval of 9 days. At each hospital visit, this researcher initially screened all patient files in the dedicated COVID-19 ward, and all patients with confirmed COVID-19 diagnosis who matched the inclusion criteria were enrolled in the study after obtaining written informed consent. Patients were assured that their personal information would remain confidential, the medical data associated with them would be anonymized, and study participation would not interfere with their treatment course in any way. The enrolled patients underwent periodontal examination and were followed up for any change in their COVID-19 severity during their hospitalization course.

### Participants

Patients were enrolled in the study from December 2021 to October 2022. Hospitalized patients with an established definitive diagnosis of COVID-19 (e.g., positive SARS-CoV-2 PCR test + clinical COVID-19 symptoms) were considered for inclusion in the study. The inclusion criteria were as follows: patients over the age of 18 with a healthy periodontium or mild gingivitis or generalized periodontitis; no history of any hereditary disease directly affecting the periodontium (e.g., Papillon-Lefèvre); no uncontrolled systemic or metabolic disease; no active neoplasm; no history of chemotherapy within the last 3 months; no history of radiotherapy within the last 6 months; no immunosuppressive therapy; and not pregnant. Exclusion criteria were as follows: systemic/metabolic disease proven to be uncontrolled; diagnosis of a new neoplasm or relapse of an old neoplasm; superinfection with other infectious agents; history of intravenous drug abuse; necrotizing periodontitis; history of comprehensive periodontal treatment within the last 6 months; periodontal examination not clinically possible; fewer than 10 teeth remaining; and withdrawal from participation.

### Periodontal examination

Patients were examined by a single periodontist using a standard periodontal probe with William’s markings (Hu-Friedy, Chicago, Illinois, US). Recorded periodontal indices were Clinical Attachment Level (CAL), Gingival Index (GI), and Modified Sulcus Bleeding Index (MSBI). These indices were measured around each tooth (excluding third molars) in 6 sites (mesiobuccal, buccal, distobuccal, mesiolingual, lingual, distolingual), and the highest measurement in each sextant was recorded. To calculate the CAL, the distance between the Cementoenamel Junction (CEJ) and the base of the periodontal pocket was measured using a probing force of 0.25 N. The GI was calculated using the original criteria proposed by Löe & Silness [19]. The MSBI, initially introduced by Mombelli et al. [20], was used to easily quantify the sulcus bleeding according to the following criteria: 0 = no bleeding; 1 = isolated points of bleeding; 2 = confluent line of bleeding on the gingival margin; 3 = heavy or profuse bleeding. The number of remaining teeth was also recorded.

### Data collection

All hematological tests were done on the attending physician’s orders. Blood samples were acquired via routine antecubital venipunctures performed by trained registered nurses. Collected blood samples were handled by hospital staff according to the ordered tests and were analyzed immediately in the hospital laboratory. The results of the hematological tests were acquired via the hospital’s information system. Tests were divided into 6 major groups: complete blood count, venous blood gas, coagulation tests, kidney and electrolytes, liver enzymes, and markers. For the suitable parameters, the maximum, minimum, and average over the entire hospitalization course were calculated. Details on the extracted test results can be found in Table 1.

**Table 1:** Extracted hematological data

Panel	Test / Parameter	Measurement Unit	Extracted Data
**Complete Blood Count (CBC)**	White Blood Cell (WBC)	Cells per microliter (#/μL)	Maximum Minimum Average
	Neutrophils	Percent (%)	
	Lymphocytes	Percent (%)	
	Red Blood Cell (RBC)	Million cells per microliter (× 10^6^/μL)	
	Hemoglobin (Hb)	Grams per deciliter (g/dL)	
	Hematocrit (Hct)	Percent (%)	
	Platelets (Plt)	Thousand cells per microliter (× 10^3^/μL)	
	Mean Corpuscular Volume (MCV)	Femtoliter (fL)	Average
	Mean Corpuscular Hemoglobin (MCH)	Picograms (pg)	
	Mean Corpuscular Hemoglobin Concentration (MCHC)	Grams per deciliter (g/dL)	
	Erythrocyte Sedimentation Rate (ESR)	Millimeters	
**Venous Blood Gas (VBG)**	Acidity (pH)	0–14 Scale	Average
	Carbon Dioxide Pressure (pCO_2_)	Millimeter of Mercury (mmHg)	
	Oxygen Pressure (pO_2_)	Millimeter of Mercury (mmHg)	
	Total Carbon Dioxide (TCO_2_)	Millimoles per liter (mmol/L)	
	Bicarbonate (HCO_3_^−^)	Millimoles per liter (mmol/L)	
	Oxygen Saturation (SO_2_)	Percent (%)	
**Coagulation**	Prothrombin Time (PT)	Seconds (s)	Maximum Minimum Average
	International Normalized Ratio (INR)	-	
	Partial Thromboplastin Time (PTT)	Seconds (s)	
**Kidney and Electrolytes**	Urea (Blood Urea Nitrogen / BUN)	Milligrams per deciliter (mg/dL)	Maximum Minimum Average
	Creatinine	Milligrams per deciliter (mg/dL)	
	Sodium (Na)	Milliequivalents per deciliter (mEq/dL)	
	Potassium (K)	Milliequivalents per deciliter (mEq/dL)	
	Calcium (Ca)	Milligrams per deciliter (mg/dL)	Average
	Phosphorous (P)	Milligrams per deciliter (mg/dL)	
	Magnesium (Mg)	Milligrams per deciliter (mg/dL)	
**Liver and Enzymes**	Aspartate Transaminase (AST / SGOT)	Units per liter (IU/L)	Maximum Minimum Average
	Alanine Transaminase (ALT / SGPT)	Units per liter (IU/L)	
	Alkaline Phosphatase (ALP)	Units per liter (IU/L)	
	Albumin	Grams per deciliter (g/dL)	Average
	Direct and Total Bilirubin	Milligrams per deciliter (mg/dL)	
**Markers**	Ferritin	Nanograms per milliliter (ng/mL)	Maximum
	Fibrin Degradation Products (FDP)	Micrograms per milliliter (μg/mL)	
	Fibrinogen	Milligrams per deciliter (mg/dL)	
	D-Dimer	Nanograms per milliliter (ng/mL)	
	Qualitative C-Reactive Protein (CRP)	-, + , + + , + + +	
	Creatin Kinase MB (CKMB)	Units per liter (IU/L)	
	Qualitative Troponins	-, +	
	Lactate Dehydrogenase (LDH)	Units per liter (IU/L)	Maximum Minimum Average

Upon conclusion of the sampling and discharge of the last enrolled patient, patient files, charts, lab results, and the hospital’s information system’s records were all extracted in image formats. All enrolled patients were followed for their discharge status and overall outcomes using the hospital’s information system. Relevant medical data were extracted. Full details regarding extracted and calculated clinical medical data can be found in the other part of the study.

### Definitions and groups

Periodontitis was defined as the presence of either 2 nonadjacent interproximal sites with detectable attachment loss or 2 nonadjacent labiolingual sites with a CAL of 3 mm or more accompanied by pocket formation [21]. Staging was performed based on the framework of the new classification of periodontal diseases [22]. Patients were divided into 3 groups based on their periodontal status:

- Healthy Periodontium (HP): healthy periodontium, localized mild gingivitis, reduced but healthy periodontium
- Mild to moderate periodontitis (MP): generalized stage I or II periodontitis
- Severe periodontitis (SP): generalized stage III or IV periodontitis

To avoid defining cases of incidental attachment loss as periodontitis, and investigate the more extensive forms of periodontal disease, we did not include patients with CAL confined to a single sextant (localized cases) in the study and only included patients who had CAL in at least two sextants accompanied by signs of inflammation such as BOP and/or an increase in the gingival index. The site with the greatest periodontal destruction was the prominent factor for determining the periodontitis stage, as described by Tonetti et al. [22], while other sites and factors, such as the number of remaining teeth, were also considered for the staging criteria that inquired about them.

COVID-19 associated organ involvement was defined as follows:

- Liver: Raise in aspartate transaminase or alanine transaminase levels to more than 3 times their normal upper limit (NUL) and/or phosphatase alkaline or bilirubin levels to more than 2 times their NUL [23].
- Kidney: ≥ 50% increase in creatinine levels compared to baseline in a 7-day period or ≥ 0.3 mg/dL increase in creatinine levels in a 48-h period [24].
- Hemato/Vascular: Disseminated Intravascular Coagulation, Deep Vein Thrombosis, Pulmonary Thromboembolism [25, 26].

COVID-19 severity grouping was done retrospectively using the following criteria based on a combination of WHO Guidelines [27] and NHC of China’s classification [28]:

- Mild to moderate COVID-19 (MC): Respiratory Symptoms, Flu-like Manifestations, Fever
- Severe to critical COVID-19 (SC): Passive Oxygenation 90% or below, Assisted Oxygenation 93% or below, Respiratory Distress, Respiratory Rate > 30, Invasive Ventilation, ICU Transfer, 50% or more Radiographic Involvement, Multiorgan Failure, Shock, Sepsis

An adverse event was defined as the occurrence of any of the following: ICU transfer, respiratory failure with subsequent need for invasive ventilation, multiorgan failure, shock, sepsis, coma, or death.

Baseline (pre-COVID-19 infection) organ deficiency/failure was defined as the following:

- Hematological: history of recent major hemorrhage, or recent disseminated intravascular coagulation, deep vein thrombosis, or pulmonary thromboembolism.
- Renal: history of end-stage renal disease, chronic kidney disease.
- Liver: history of cirrhosis, fatty liver, or liver surgery.
- Pulmonary: history of asthma, emphysema, or chronic obstructive pulmonary disease.

An adverse event was defined as the occurrence of any of the following: ICU Transfer, Respiratory Failure with subsequent need for invasive ventilation, Multi-organ Failure, Shock, Sepsis, Coma, Death.

### Confounders and covariates

All available data pertaining to possible covariates/confounders such as age, sex, smoking, comorbidities, and medications were collected. Smoking status was categorized into current smokers, former smokers, and nonsmokers. Not enough standardized data were available to quantize smoking status for all patients in the form of pack-years or cigarettes/day, nor for body mass index or socioeconomic status.

Comorbidities were divided into 9 major categories based on all patient's medical histories: hypertension, coronary artery disease (ischemic heart disease, myocardial infarction, etc.), congestive heart failure, respiratory disorders (chronic obstructive pulmonary disease, asthma, bronchiectasis), renal disease (end-stage renal disease, chronic kidney disease, etc.), diabetes mellitus, autoimmune disorders (systemic lupus erythematous, arthritis rheumatoid, multiple sclerosis, etc.), chronic liver disease, and cancer in remission.

Medications were divided into principal COVID-19 medications, baseline-condition medications (such as medications for hypertension), and other medications (such as analgesics, antiacids, and laxatives). Principal COVID-19 medications included anticoagulants (e.g., enoxaparin, rivaroxaban), antiplatelets (e.g., clopidogrel), corticosteroids (e.g., dexamethasone, prednisolone), antivirals (e.g., remdesivir), antibiotics (e.g., meropenem, ceftriaxone), bronchodilators (e.g., salbutamol, ipratropium), and cytokine inhibitors (e.g., tocilizumab).

### Bias reduction, sample size calculation, data analysis

To reduce examination and data collection bias, the periodontal examiner was blinded to the medical information of the patient, and the medical file screener was blinded to the oral status of the patient (i.e., the periodontal examiner had no knowledge about the patient’s COVID-19 severity, and the medical file screener had no knowledge regarding the patient’s periodontal status). All patients were followed up using the hospital’s information system in a single session at the conclusion of the study. No matching was performed as all cases and controls were included in the analyses.

This study and its analyses on hematological tests are a part of a greater study in which the clinical parameters of COVID-19, periodontal status and indices, salivary and serum interleukin-6 (IL-6) and C-reactive protein (CRP), and other related parameters and their association were investigated, and thus shares the same sample pool with that study. Since there was no similar published study at the time of conception of that study, the sample size was calculated using a previous study on the association between periodontitis and IL-6 levels [29]. Considering an expected mean difference of 0.7 pg/dL in levels of IL-6 between different periodontitis severity groups and a variance of 0.8 pg/dL, the minimum sample size for a power of 80% and an alpha value of 5% was calculated to be 120 in total (average of 20 patients in each of the 6 subgroups). After data collection, analysis was performed using IBM SPSS Statistics v26 (IBM Corp., New York, USA; RRID:SCR_019096).

Potential confounders were determined to be age, sex and comorbidities. Smoking status had no statistically significant difference across COVID-19 or periodontal severity groups and had missing data. Treatment medications had no statistically significant difference across COVID-19 or periodontal severity groups. There were not enough available data for body mass index or socioeconomic status.

To mitigate the pronounced effect of baseline medical comorbidities on some tests, the following were done:

- Patients with baseline hematological deficiency/failure were excluded from complete blood count (CBC) and coagulation analyses.
- Patients with baseline renal deficiency/failure were excluded from urea, creatinine, and electrolytes analyses.
- Patients with baseline liver deficiency/failure were excluded from liver enzyme and bilirubin analyses.
- In patients with baseline pulmonary deficiency/failure, a lower threshold for normal oxygen saturation was considered compared to healthy individuals, based on the recommendations from the WHO guideline [27].

Age was used as a continuous variable in the analyses, while sex and comorbidities were used as binary variables. Critical parameters were the severity assessments of COVID-19 and periodontitis. All patients had severity assessments for both diseases. The maximum acceptable missing value percentage for analyses of hematological parameters was set to 20%.

The results for continuous variables were reported as the mean ± standard deviation, and those for categorical variables were reported as frequency and percentage. To assess the normality of distributions in the data, the Kolmogorov–Smirnov test was used. ANCOVA was used to compare continuous variables with roughly normal distributions across severity groups while accounting for confounders and covariates and to investigate the possible interaction between the two diseases.

## Results

### Participants

**Fig. 1:**
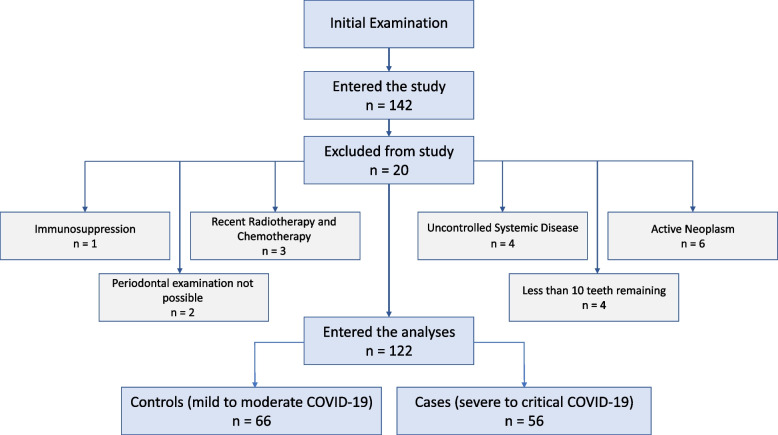
Enrollment flowchart

After the initial examination, 142 patients were included in the study. Upon the conclusion of follow-ups and a thorough examination of medical records, 20 patients were excluded from the study. The diagram for study enrollment is presented in Fig. 1.

### General results

Age had a normal distribution and a mean of 52.82 ± 13.34 years, with the youngest patient being 19 and the oldest being 78 years old. A total of 51.6% (*n* = 63) of patients were male. Length of stay (LOS) did not have a normal distribution. The longest LOS was 71 days, and the shortest LOS was 3 days. The mean, median, and interquartile range for LOS were 10.24, 8, and 5 days, respectively. Of all patients, 27 (22.1%) were transferred to the intensive care unit (ICU) during their hospitalization course. The mean oxygen saturation with passive oxygenation for the last three records was 90.82 ± 4.83%. The mean lung involvement% in CT scans was 35.97 ± 16.83%, with the most prevalent appearance being ground glass opacities. The most common findings in medical history were hypertension (33.6%, *n* = 41), diabetes mellitus (13.1%, *n* = 16) and chronic obstructive pulmonary disease (10.7%, *n* = 13). Of the patients with available smoking history (*n* = 111), 27% (*n* = 30) of patients were current smokers, while 2.5% (*n* = 3) were former smokers. A total of 7.4% (*n* = 9) of patients had respiratory distress initially or during their hospitalization. COVID-19 associated organ involvement was found in 23% (*n* = 28) of patients, with liver involvement being the most prevalent (13.9% of all patients, *n* = 17). Adverse events occurred in 27.9% (n = 34) of patients, including 3 deaths. The general and medical data can be found in Tables 2 and 3.

**Table 2:** General data

**Variable**	**n**^**a**^	**Subcategory**	**Frequency (Percent)**			
**Sex**	122	*Male*	63 (51.6%)			
		*Female*	59 (48.4%)			
**Survival**	122	*Survived*	119 (97.5%)			
		*Deceased*	3 (2.5%)			
**Variable**	**n**	**Mean ± Standard Deviation**	**Median**	**Min**	**Max**	**Range**
**Age** (years)	122	52.82 ± 13.34	54	19	78	59
**Hospitalization Length** (days)	122	10.24 ± 8.40	8	3	71	68

^a^Number of available data

**Table 3:** General medical data

**Variable**	**Subcategory**	**n**^**a**^	**Average ± SD**^**b**^	**Min**		**Max**	**Range**
**Temperature** (Celsius)	*Max*	110	37.23 ± 0.47	36.5		40	3.5
	*Min*	106	36.27 ± 0.46	34.2		37.4	3.2
	*L3A*^c^	106	36.69 ± 0.26	36.2		37.4	1.2
**Blood Pressure** (mmHg)	*Systolic L3A*	113	114.88 ± 12.35	93.33		150	56.7
	*Diastolic L3A*	113	71.86 ± 9.11	53.33		110	56.7
**Respiratory Rate** (per minute)	*Max*	107	21.07 ± 4.76	15		46	31
	*L3A*	105	18.01 ± 1.09	15		20.5	5.5
**Oxygen Saturation** (%)	*Assisted Oxygenation L3A*	81	95.28 ± 2.85	81		99.7	18.7
	*Passive Oxygenation Max*	101	93.33 ± 3.71	82		100	18
	*Passive Oxygenation Min*	106	86.18 ± 7.2	52		98	46
	*Passive Oxygenation L3A*	99	90.82 ± 4.83	73		98	25
	*Baseline*	111	85.44 ± 10.85	42		98	56
**CT Scan Lung Involvement**	*Extent (%)*	119	35.97 ± 16.83	5		75	70
**Variable**	**Subcategory**			n	Count		Ratio (%)^d^
**CT Scan Lung Involvement Appearance**	*Ground Glass Opacities (GGO)*			119	63		52.9
	*Consolidations*				7		5.9
	*Atelectasis*				5		4.2
	*Nodular*				4		3.4
	*Embolic*				1		0.8
	*GGO* + *Consolidations*				18		15.1
	*GGO* + *Atelectasis*				9		7.6
	*GGO* + *Embolic*				8		6.7
	*GGO* + *Nodular*				4		3.4
**Significant Medical History Items**	*Hypertension*			122	41		33.6
	*Diabetes Mellitus*				16		13.1
	*Neoplasm (in remission)*				14		11.5
	*Chronic Obstructive Pulmonary Disease*				13		10.7
	*End-Stage Renal disease*				10		8.2
	*Coronary Artery Disease*				10		8.2
	*Autoimmune Disorder*				10		8.2
	*Hypothyroidism*				7		5.7
	*Asthma*				6		4.9
	*Cerebrovascular Accident*				5		4.1
	*Chronic Kidney Disease*				4		3.3
**Smoking**	*Yes*			111	30		27
	*Former*				3		2.7
	*No*				78		70.3
**COVID-19 Associated Organ Involvement**	*Liver*			122	17		13.9
	*Hemato/Vascular*				10		8.2
	*Kidney*				7		5.7
	*Multiple Organs*				8		6.5
**ICU Admission**	*Yes*			122	27		22.1
	*No*				95		77.9
**Adverse Event**	*Yes*			122	34		27.9
	*No*				88		72.1
**COVID-19 Severity**	*Mild to Moderate*			122	66		54.1
	*Severe to Critical*				56		45.9
**Periodontitis Severity**	*Healthy / Localized Mild Gingivitis*			122	37		30.3
	*Generalized Stage I or II*				42		34.4
	*Generalized Stage III or IV*				43		35.2

^a^Number of available data

^b^Standard Deviation

^c^Last three records’ average

^d^Percentage from available data(n)

### Hematological tests

**Fig. 2:**
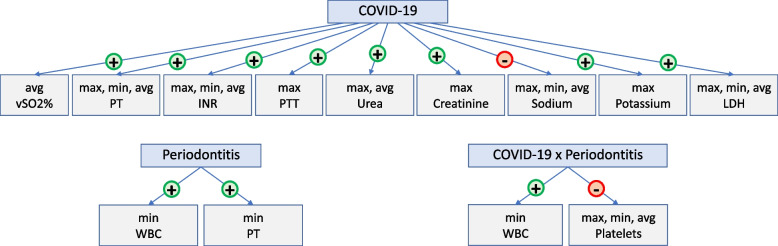
Summary of significant associations. Positive green circle: Significant positive association; with an increase in disease severity, the parameter was increased. Negative red circle: Significant negative association; with an increase in disease severity, the parameter was decreased

For all analyses, age, sex, and comorbidities were considered as confounders. ANCOVA was used to evaluate associations between test results, COVID-19, and periodontitis. Full details of all hematological analyses can be found in Table 4. A schematic representation of all statistically significant associations in Table 4 can be found in Fig. 2.

**Table 4:** Hematological data

Variable	COVID-19 Severity	Mean ± Standard Deviation (number of available data)				ANCOVA^a^	
		**Periodontitis Severity Group**				**Element **^b^	***P*** **Value**
		*HP*	*MP*	*SP*	*Total*		
**Complete Blood Count (patients excluded = 4)**							
**Max WBC** (#/μL)	*MC*	9156 ± 4468 (27)	10,292 ± 4922 (26)	14,809 ± 3260 (11)	10,589 ± 4855 (64)	COVID-19	0.178
	*SC*	14,170 ± 8518 (10)	11,177 ± 4746 (13)	13,600 ± 5751 (31)	13,122 ± 6116 (54)	Periodontitis	0.116
	*Total*	10,511 ± 6136 (37)	10,587 ± 4820 (42)	13,917 ± 5204 (43)	11,748 ± 5589 (118)	COVID-19 x Periodontitis	0.161
**Min WBC** (#/μL)	*MC*	6556 ± 2864 (27)	6077 ± 2075 (26)	10,209 ± 4487 (11)	6989 ± 3244 (64)	COVID-19	0.273
	*SC*	9650 ± 5653 (10)	7369 ± 3705 (13)	8297 ± 3631 (31)	8324 ± 4068 (54)	**Periodontitis**	0.037 *
	*Total*	7392 ± 3982 (37)	6508 ± 2747 (42)	8798 ± 3909 (43)	7600 ± 3689 (118)	**COVID-19** **x** **Periodontitis**	0.032 ^*^
**Average WBC** (#/μL)	*MC*	7822 ± 3398 (27)	8230 ± 3222 (26)	12,388 ± 3718 (11)	8772 ± 3724 (64)	COVID-19	0.174
	*SC*	11,900 ± 6843 (10)	9189 ± 3845 (13)	10,779 ± 4223 (31)	10,604 ± 4714 (54)	Periodontitis	0.065
	*Total*	8924 ± 4839 (37)	8550 ± 3422 (42)	11,200 ± 4115 (43)	9610 ± 4286 (118)	COVID-19 x Periodontitis	0.059
**Max Neutrophils** (%)	*MC*	81.76 ± 12.96 (27)	83.50 ± 7.76 (26)	87.64 ± 4.41 (11)	83.48 ± 10.03 (64)	COVID-19	0.409
	*SC*	87.00 ± 8.78 (10)	84.46 ± 8.69 (13)	87.13 ± 9.67 (31)	86.46 ± 9.19 (54)	Periodontitis	0.545
	*Total*	83.17 ± 12.09 (37)	83.82 ± 7.98 (42)	87.26 ± 8.56 (43)	84.84 ± 9.73 (118)	COVID-19 x Periodontitis	0.653
**Min Neutrophils** (%)	*MC*	70.83 ± 14.03 (27)	72.38 ± 7.78 (26)	73.82 ± 13.17 (11)	71.97 ± 11.57 (64)	COVID-19	0.492
	*SC*	77.80 ± 8.00 (10)	70.38 ± 11.27 (13)	73.32 ± 11.16 (31)	73.44 ± 10.77 (54)	Periodontitis	0.678
	*Total*	72.71 ± 12.96 (37)	71.72 ± 8.99 (42)	73.45 ± 11.55 (43)	72.65 ± 11.19 (118)	COVID-19 x Periodontitis	0.237
**Average Neutrophils** (%)	*MC*	76.96 ± 12.30 (27)	78.21 ± 7.02 (26)	80.54 ± 6.95 (11)	78.09 ± 9.55 (64)	COVID-19	0.256
	*SC*	82.22 ± 8.27 (10)	78.90 ± 8.51 (13)	81.36 ± 8.35 (31)	80.92 ± 8.30 (54)	Periodontitis	0.695
	*Total*	78.38 ± 11.49 (37)	78.44 ± 7.44 (42)	81.14 ± 7.93 (43)	79.38 ± 9.08 (118)	COVID-19 x Periodontitis	0.633
**Max Lymphocytes** (%)	*MC*	22.89 ± 13.62 (27)	21.92 ± 7.04 (26)	19.64 ± 8.73 (11)	21.94 ± 10.47 (64)	COVID-19	0.275
	*SC*	16.30 ± 8.55 (10)	22.69 ± 9.79 (13)	18.68 ± 8.55 (31)	19.20 ± 8.96 (54)	Periodontitis	0.442
	*Total*	21.11 ± 12.69 (37)	22.18 ± 7.94 (42)	18.93 ± 8.50 (43)	20.69 ± 9.86 (118)	COVID-19 x Periodontitis	0.287
**Min Lymphocytes** (%)	*MC*	14.03 ± 11.10 (27)	12.77 ± 6.20 (26)	9.00 ± 3.79 (11)	12.65 ± 8.46 (64)	COVID-19	0.485
	*SC*	9.60 ± 7.63 (10)	12.69 ± 7.42 (13)	9.26 ± 8.44 (31)	10.15 ± 8.05 (54)	Periodontitis	0.365
	*Total*	12.84 ± 10.37 (37)	12.74 ± 6.53 (42)	9.19 ± 7.46 (43)	11.51 ± 8.33 (118)	COVID-19 x Periodontitis	0.598
**Average Lymphocytes** (%)	*MC*	18.39 ± 11.96 (27)	17.15 ± 5.64 (26)	14.22 ± 4.65 (11)	17.17 ± 8.79 (64)	COVID-19	0.234
	*SC*	12.95 ± 8.15 (10)	16.59 ± 7.67 (13)	13.69 ± 7.55 (31)	14.25 ± 7.66 (54)	Periodontitis	0.467
	*Total*	16.92 ± 11.22 (37)	16.97 ± 6.29 (42)	13.83 ± 6.86 (43)	15.84 ± 8.39 (118)	COVID-19 x Periodontitis	0.467
**Max RBC** (× 10^6^/μL)	*MC*	4.47 ± 0.95 (27)	4.72 ± 0.85 (26)	4.92 ± 1.00 (11)	4.65 ± 0.92 (64)	COVID-19	0.952
	*SC*	4.65 ± 0.99 (10)	4.71 ± 1.12 (13)	4.72 ± 1.09 (31)	4.70 ± 1.06 (54)	Periodontitis	0.982
	*Total*	4.52 ± 0.95 (37)	4.72 ± 0.93 (42)	4.77 ± 1.06 (43)	4.67 ± 0.98 (118)	COVID-19 x Periodontitis	0.933
**Min RBC** (× 10^6^/μL)	*MC*	4.16 ± 0.90 (27)	4.13 ± 0.75 (26)	4.34 ± 1.10 (11)	4.18 ± 0.87 (64)	COVID-19	0.456
	*SC*	4.04 ± 1.00 (10)	4.16 ± 1.26 (13)	3.99 ± 1.21 (31)	4.04 ± 1.17 (54)	Periodontitis	0.862
	*Total*	4.13 ± 0.92 (37)	4.14 ± 0.93 (42)	4.09 ± 1.18 (43)	4.12 ± 1.01 (118)	COVID-19 x Periodontitis	0.844
**Average RBC** (× 10^6^/μL)	*MC*	4.31 ± 0.92 (27)	4.39 ± 0.75 (26)	4.61 ± 1.02 (11)	4.40 ± 0.86 (64)	COVID-19	0.671
	*SC*	4.35 ± 0.92 (10)	4.41 ± 1.15 (13)	4.35 ± 1.08 (31)	4.36 ± 1.05 (54)	Periodontitis	0.884
	*Total*	4.32 ± 0.90 (37)	4.40 ± 0.89 (42)	4.42 ± 1.06 (43)	4.38 ± 0.95 (118)	COVID-19 x Periodontitis	0.925
**Max Hemoglobin** (g/dL)	*MC*	12.53 ± 2.80 (27)	13.55 ± 2.52 (26)	14.29 ± 2.89 (11)	13.25 ± 2.75 (64)	COVID-19	0.552
	*SC*	13.05 ± 2.88 (10)	13.05 ± 2.27 (13)	13.25 ± 3.15 (31)	13.17 ± 2.86 (54)	Periodontitis	0.949
	*Total*	12.67 ± 2.79 (37)	13.39 ± 2.42 (42)	13.52 ± 3.08 (43)	13.21 ± 2.79 (118)	COVID-19 x Periodontitis	0.854
**Min Hemoglobin** (g/dL)	*MC*	11.66 ± 2.66 (27)	11.83 ± 2.12 (26)	12.29 ± 2.85 (11)	11.83 ± 2.46 (64)	COVID-19	0.164
	*SC*	11.11 ± 2.97 (10)	11.32 ± 2.74 (13)	11.00 ± 3.27 (31)	11.10 ± 3.05 (54)	Periodontitis	0.708
	*Total*	11.51 ± 2.72 (37)	11.66 ± 2.32 (42)	11.34 ± 3.18 (43)	11.50 ± 2.76 (118)	COVID-19 x Periodontitis	0.940
**Average Hemoglobin** (g/dL)	*MC*	12.09 ± 2.72 (27)	12.57 ± 2.17 (26)	13.22 ± 2.66 (11)	12.48 ± 2.49 (64)	COVID-19	0.298
	*SC*	12.09 ± 2.67 (10)	12.11 ± 2.30 (13)	12.10 ± 3.00 (31)	12.10 ± 2.74 (54)	Periodontitis	0.769
	*Total*	12.09 ± 2.67 (37)	12.42 ± 2.20 (42)	12.39 ± 2.93 (43)	12.31 ± 2.60 (118)	COVID-19 x Periodontitis	0.957
**Max Hematocrit** (%)	*MC*	38.40 ± 8.07 (27)	41.57 ± 6.99 (26)	43.21 ± 7.64 (11)	40.51 ± 7.69 (64)	COVID-19	0.684
	*SC*	39.60 ± 7.87 (10)	40.67 ± 7.93 (13)	40.80 ± 9.65 (31)	40.55 ± 8.81 (54)	Periodontitis	0.973
	*Total*	38.73 ± 7.92 (37)	41.27 ± 7.23 (42)	41.43 ± 9.14 (43)	40.53 ± 8.19 (118)	COVID-19 x Periodontitis	0.948
**Min Hematocrit** (%)	*MC*	36.08 ± 7.64 (27)	36.33 ± 6.41 (26)	38.24 ± 8.18 (11)	36.55 ± 7.18 (64)	COVID-19	0.309
	*SC*	35.31 ± 9.47 (10)	35.95 ± 8.91 (13)	34.42 ± 10.04 (31)	34.95 ± 9.52 (54)	Periodontitis	0.672
	*Total*	35.87 ± 8.04 (37)	36.20 ± 7.22 (42)	35.42 ± 9.64 (43)	35.82 ± 8.34 (118)	COVID-19 x Periodontitis	0.843
**Average Hematocrit** (%)	*MC*	36.86 ± 8.18 (27)	38.59 ± 6.37 (26)	37.80 ± 9.50 (11)	37.73 ± 7.66 (64)	COVID-19	0.899
	*SC*	36.96 ± 7.41 (10)	38.14 ± 7.91 (13)	37.54 ± 9.20 (31)	37.58 ± 8.46 (54)	Periodontitis	0.568
	*Total*	36.89 ± 7.88 (37)	38.44 ± 6.82 (42)	37.61 ± 9.16 (43)	37.66 ± 8.00 (118)	COVID-19 x Periodontitis	0.913
**Max Platelets** (× 10^3^/μL)	*MC*	216.3 ± 62.9 (27)	227.3 ± 80.9 (26)	300.7 ± 115.2 (11)	235.3 ± 85.2 (64)	COVID-19	0.315
	*SC*	251.3 ± 60.2 (10)	224.2 ± 107.3 (13)	213.3 ± 100.6 (31)	222.9 ± 95.7 (54)	Periodontitis	0.114
	*Total*	225.8 ± 63.3 (37)	226.3 ± 89.1 (42)	236.2 ± 110.2 (43)	229.6 ± 90.0 (118)	**COVID-19** **x** **Periodontitis**	0.008 ^*^
**Min Platelets** (× 10^3^/μL)	*MC*	176.7 ± 65.9 (27)	165.0 ± 56.8 (26)	239.2 ± 94.7 (11)	182.7 ± 72.1 (64)	COVID-19	0.150
	*SC*	206.4 ± 40.8 (10)	162.2 ± 91.6 (13)	148.2 ± 89.9 (31)	162.4 ± 85.1 (54)	Periodontitis	0.068
	*Total*	184.8 ± 61.1 (37)	164.1 ± 69.1 (42)	172.0 ± 98.7 (43)	173.4 ± 78.6 (118)	**COVID-19** **x** **Periodontitis**	0.001 ^*^
**Average Platelets** (× 10^3^/μL)	*MC*	196.37 ± 61.80 (27)	197.41 ± 62.80 (26)	270.61 ± 100.41 (11)	209.55 ± 74.35 (64)	COVID-19	0.164
	*SC*	226.03 ± 42.60 (10)	192.56 ± 95.46 (13)	178.46 ± 91.41 (31)	190.66 ± 86.17 (54)	Periodontitis	0.085
	*Total*	204.39 ± 58.23 (37)	195.79 ± 74.01 (42)	202.59 ± 101.26 (43)	200.91 ± 80.18 (118)	**COVID-19** **x** **Periodontitis**	0.002 ^*^
**MCV** (fL)	*MC*	86.55 ± 5.02 (27)	88.36 ± 6.85 (26)	89.03 ± 8.52 (11)	87.71 ± 6.45 (64)	COVID-19	0.348
	*SC*	85.19 ± 3.97 (10)	87.29 ± 4.55 (13)	86.88 ± 9.74 (31)	86.67 ± 7.85 (54)	Periodontitis	0.760
	*Total*	86.18 ± 4.74 (37)	88.00 ± 6.14 (42)	87.45 ± 9.38 (43)	87.23 ± 7.11 (118)	COVID-19 x Periodontitis	0.945
**MCH** (pg)	*MC*	28.13 ± 1.80 (27)	28.75 ± 2.80 (26)	28.89 ± 3.65 (11)	28.51 ± 2.59 (64)	COVID-19	0.273
	*SC*	27.74 ± 1.98 (10)	27.79 ± 1.88 (13)	28.05 ± 4.32 (31)	27.93 ± 3.47 (54)	Periodontitis	0.903
	*Total*	28.02 ± 1.83 (37)	28.43 ± 2.55 (42)	28.27 ± 4.13 (43)	28.24 ± 3.02 (118)	COVID-19 x Periodontitis	0.947
**MCHC** (g/dL)	*MC*	32.38 ± 1.28 (27)	32.44 ± 1.44 (26)	32.39 ± 1.80 (11)	32.41 ± 1.42 (64)	COVID-19	0.439
	*SC*	32.55 ± 1.54 (10)	31.81 ± 1.20 (13)	32.14 ± 1.75 (31)	32.14 ± 1.59 (54)	Periodontitis	0.375
	*Total*	32.43 ± 1.34 (37)	32.23 ± 1.38 (42)	32.21 ± 1.74 (43)	32.28 ± 1.50 (118)	COVID-19 x Periodontitis	0.637
**Average ESR** (mm)	*MC*	48.35 ± 36.62 (24)	42.62 ± 30.90 (25)	46.83 ± 37.72 (9)	45.64 ± 33.91 (58)	COVID-19	0.230
	*SC*	63.70 ± 34.80 (10)	54.30 ± 46.14 (13)	48.16 ± 37.38 (30)	52.604 ± 38.94 (53)	Periodontitis	0.965
	*Total*	52.86 ± 36.27 (34)	46.61 ± 36.62 (28)	47.85 ± 36.96 (39)	48.96 ± 36.40 (111)	COVID-19 x Periodontitis	0.570
**Venous Blood Gas**							
**pH**	*MC*	7.401 ± 0.086 (24)	7.394 ± 0.053 (26)	7.379 ± 0.060 (9)	7.394 ± 0.069 (59)	COVID-19	0.972
	*SC*	7.382 ± 0.046 (8)	7.412 ± 0.046 (14)	7.392 ± 0.084 (29)	7.396 ± 0.070 (51)	Periodontitis	0.872
	*Total*	7.396 ± 0.078 (32)	7.400 ± 0.051 (40)	7.389 ± 0.079 (38)	7.395 ± 0.069 (110)	COVID-19 x Periodontitis	0.707
**pCO**_**2**_ (mmHg)	*MC*	39.61 ± 11.15 (24)	36.98 ± 14.56 (26)	39.87 ± 7.62 (9)	38.49 ± 12.27 (59)	COVID-19	0.619
	*SC*	35.99 ± 10.17 (8)	35.57 ± 9.66 (14)	39.43 ± 9.99 (29)	37.83 ± 9.90 (51)	Periodontitis	0.821
	*Total*	38.71 ± 10.87 (32)	36.49 ± 12.94 (40)	39.53 ± 9.38 (38)	38.18 ± 11.19 (110)	COVID-19 x Periodontitis	0.665
**pO**_**2**_ (mmHg)	*MC*	31.48 ± 12.88 (24)	37.53 ± 12.80 (26)	36.14 ± 14.96 (9)	34.86 ± 13.25 (59)	**COVID-19**	0.022 ^*^
	*SC*	39.04 ± 10.52 (8)	52.56 ± 25.50 (14)	41.16 ± 17.39 (29)	43.96 ± 19.57 (51)	Periodontitis	0.108
	*Total*	33.37 ± 12.62 (32)	42.79 ± 19.35 (40)	39.97 ± 16.79 (38)	39.08 ± 17.03 (110)	COVID-19 x Periodontitis	0.452
**TCO**_**2**_ (mmol/L)	*MC*	25.32 ± 4.49 (24)	23.22 ± 7.80 (26)	24.75 ± 3.87 (9)	24.31 ± 6.11 (59)	COVID-19	0.614
	*SC*	22.57 ± 6.22 (8)	23.38 ± 6.04 (13)	25.28 ± 6.44 (29)	24.35 ± 6.28 (50)	Periodontitis	0.739
	*Total*	24.63 ± 5.02 (32)	23.27 ± 7.18 (39)	25.15 ± 5.89 (38)	24.33 ± 6.16 (109)	COVID-19 x Periodontitis	0.476
**HCO**_**3**_^**−**^ (mmol/L)	*MC*	24.13 ± 4.28 (24)	22.08 ± 7.39 (26)	23.53 ± 3.72 (9)	23.13 ± 5.80 (59)	COVID-19	0.630
	*SC*	21.53 ± 6.00 (8)	22.29 ± 5.84 (13)	24.09 ± 6.24 (29)	23.21 ± 6.07 (50)	Periodontitis	0.729
	*Total*	23.48 ± 4.80 (32)	22.15 ± 6.84 (39)	23.95 ± 5.70 (38)	23.17 ± 5.90 (109)	COVID-19 x Periodontitis	0.476
**SO**_**2**_ (%)	*MC*	64.28 ± 15.48 (18)	67.81 ± 15.63 (24)	72.96 ± 14.63 (7)	67.25 ± 15.39 (49)	COVID-19	0.191
	*SC*	68.18 ± 17.69 (8)	79.72 ± 11.38 (13)	75.18 ± 14.76 (25)	75.25 ± 14.63 (46)	Periodontitis	0.418
	*Total*	65.48 ± 15.93 (26)	72.00 ± 15.24 (37)	74.70 ± 14.53 (32)	71.12 ± 15.48 (95)	COVID-19 x Periodontitis	0.422
**Coagulation (patients excluded = 4)**							
**Max PT** (s)	*MC*	13.19 ± 2.17 (26)	13.15 ± 1.92 (26)	13.44 ± 1.75 (10)	13.22 ± 1.98 (62)	**COVID-19**	0.013 ^*^
	*SC*	18.60 ± 16.59 (10)	13.65 ± 1.92 (13)	18.87 ± 10.26 (30)	17.54 ± 10.59 (53)	Periodontitis	0.089
	*Total*	14.69 ± 8.95 (36)	13.32 ± 1.91 (39)	17.51 ± 9.20 (40)	15.21 ± 7.61 (115)	COVID-19 x Periodontitis	0.276
**Min PT** (s)	*MC*	12.91 ± 1.36 (26)	12.99 ± 1.45 (26)	13.44 ± 1.75 (10)	13.03 ± 1.45 (62)	**COVID-19**	0.016^*^
	*SC*	15.67 ± 8.34 (10)	12.88 ± 1.37 (13)	16.03 ± 5.06 (30)	15.19 ± 5.34 (53)	**Periodontitis**	0.047 ^*^
	*Total*	13.68 ± 4.56 (36)	12.95 ± 1.41 (39)	15.38 ± 4.58 (40)	14.03 ± 3.91 (115)	COVID-19 x Periodontitis	0.204
**Average PT** (s)	*MC*	13.07 ± 1.76 (26)	13.06 ± 1.62 (26)	13.44 ± 1.75 (10)	13.13 ± 1.68 (62)	**COVID-19**	0.010 ^*^
	*SC*	17.16 ± 12.54 (10)	13.22 ± 1.47 (13)	17.15 ± 6.60 (30)	16.19 ± 7.41 (53)	Periodontitis	0.056
	*Total*	14.21 ± 6.79 (36)	13.11 ± 1.55 (39)	16.22 ± 5.98 (40)	14.54 ± 5.38 (115)	COVID-19 x Periodontitis	0.214
**Max INR**	*MC*	1.148 ± 0.193 (26)	1.140 ± 0.174 (26)	1.170 ± 0.134 (10)	1.148 ± 0.174 (62)	**COVID-19**	0.014 ^*^
	*SC*	1.749 ± 1.953 (10)	1.195 ± 0.192 (13)	1.656 ± 0.934 (30)	1.561 ± 1.096 (53)	Periodontitis	0.114
	*Total*	1.315 ± 1.040 (36)	1.158 ± 0.180 (39)	1.535 ± 0.836 (40)	1.338 ± 0.779 (115)	COVID-19 x Periodontitis	0.280
**Min INR**	*MC*	1.116 ± 0.094 (26)	1.121 ± 0.119 (26)	1.170 ± 0.134 (10)	1.127 ± 0.111 (62)	**COVID-19**	0.026 ^*^
	*SC*	1.391 ± 0.918 (10)	1.122 ± 0.114 (13)	1.397 ± 0.510 (30)	1.328 ± 0.555 (53)	Periodontitis	0.089
	*Total*	1.193 ± 0.489 (36)	1.121 ± 0.116 (39)	1.340 ± 0.455 (40)	1.220 ± 0.397 (115)	COVID-19 x Periodontitis	0.288
**Average INR**	*MC*	1.135 ± 0.146 (26)	1.129 ± 0.135 (26)	1.169 ± 0.134 (10)	1.138 ± 0.138 (62)	**COVID-19**	0.014 ^*^
	*SC*	1.555 ± 1.387 (10)	1.153 ± 0.138 (13)	1.503 ± 0.637 (30)	1.427 ± 0.767 (53)	Periodontitis	0.089
	*Total*	1.252 ± 0.739 (36)	1.137 ± 0.135 (39)	1.420 ± 0.572 (40)	1.271 ± 0.548 (115)	COVID-19 x Periodontitis	0.260
**Max PTT** (s)	*MC*	29.63 ± 5.03 (26)	31.22 ± 14.41 (26)	29.82 ± 4.54 (10)	30.33 ± 9.95 (62)	**COVID-19**	0.016 ^*^
	*SC*	39.87 ± 24.57 (10)	37.05 ± 17.12 (13)	37.80 ± 20.60 (30)	38.00 ± 20.24 (53)	Periodontitis	0.896
	*Total*	32.48 ± 13.96 (36)	33.16 ± 15.39 (39)	35.80 ± 18.23 (40)	33.87 ± 15.96 (115)	COVID-19 x Periodontitis	0.885
**Min PTT** (s)	*MC*	29.33 ± 4.95 (26)	30.55 ± 14.40 (26)	29.82 ± 4.54 (10)	29.92 ± 9.92 (62)	COVID-19	0.480
	*SC*	30.76 ± 5.12 (10)	32.22 ± 12.14 (13)	29.34 ± 5.97 (30)	30.32 ± 7.74 (53)	Periodontitis	0.572
	*Total*	29.73 ± 4.96 (36)	31.10 ± 13.55 (39)	29.46 ± 5.60 (40)	30.10 ± 8.95 (115)	COVID-19 x Periodontitis	0.911
**Average PTT** (s)	*MC*	29.48 ± 4.95 (26)	30.89 ± 14.31 (26)	29.82 ± 4.54 (10)	30.13 ± 9.87 (62)	COVID-19	0.054
	*SC*	35.26 ± 12.16 (10)	34.10 ± 12.14 (13)	32.63 ± 9.84 (30)	33.49 ± 10.71 (53)	Periodontitis	0.809
	*Total*	31.09 ± 7.90 (36)	31.96 ± 13.55 (39)	31.93 ± 8.85 (40)	31.67 ± 10.36 (115)	COVID-19 x Periodontitis	0.893
**Kidney and Electrolytes (patients excluded = 16)**							
**Max Urea** (mg/dL)	*MC*	32.48 ± 7.72 (23)	38.92 ± 12.94 (24)	44.18 ± 12.34 (11)	37.36 ± 11.70 (58)	**COVID-19**	< 0.001 ^*^
	*SC*	65.67 ± 55.31 (9)	51.83 ± 30.91 (12)	68.26 ± 35.48 (27)	63.67 ± 38.59 (48)	Periodontitis	0.243
	*Total*	41.81 ± 32.58 (32)	43.22 ± 21.18 (36)	61.29 ± 32.37 (38)	49.27 ± 30.23 (106)	COVID-19 x Periodontitis	0.436
**Min Urea** (mg/dL)	*MC*	25.65 ± 6.14 (23)	26.21 ± 10.53 (24)	36.18 ± 10.86 (11)	27.88 ± 9.82 (58)	COVID-19	0.098
	*SC*	41.67 ± 42.69 (9)	29.83 ± 10.27 (12)	36.56 ± 18.81 (27)	35.83 ± 23.38 (48)	Periodontitis	0.161
	*Total*	30.16 ± 23.47 (32)	27.42 ± 10.44 (36)	36.45 ± 16.75 (38)	31.48 ± 17.69 (106)	COVID-19 x Periodontitis	0.280
**Average Urea** (mg/dL)	*MC*	29.24 ± 6.29 (23)	32.22 ± 11.05 (24)	40.24 ± 10.49 (11)	32.56 ± 9.98 (58)	**COVID-19**	0.003 ^*^
	*SC*	54.52 ± 51.50 (9)	38.65 ± 13.60 (12)	51.22 ± 24.61 (27)	48.69 ± 29.42 (48)	Periodontitis	0.122
	*Total*	36.35 ± 29.08 (32)	34.36 ± 12.16 (36)	48.04 ± 21.92 (38)	39.87 ± 22.51 (106)	COVID-19 x Periodontitis	0.272
**Max Creatinine** (mg/dL)	*MC*	0.974 ± 0.171 (23)	1.067 ± 0.199 (24)	1.182 ± 0.322 (11)	1.052 ± 0.227 (58)	**COVID-19**	0.042 ^*^
	*SC*	1.778 ± 2.270 (9)	1.104 ± 0.179 (12)	1.467 ± 1.042 (27)	1.435 ± 1.239 (48)	Periodontitis	0.349
	*Total*	1.200 ± 1.219 (32)	1.079 ± 0.191 (36)	1.384 ± 0.899 (38)	1.225 ± 0.867 (106)	COVID-19 x Periodontitis	0.276
**Min Creatinine** (mg/dL)	*MC*	0.852 ± 0.165 (23)	0.859 ± 0.174 (24)	0.964 ± 0.186 (11)	0.876 ± 0.175 (58)	COVID-19	0.121
	*SC*	1.356 ± 1.744 (9)	0.892 ± 0.183 (12)	0.999 ± 0.479 (27)	1.039 ± 0.824 (48)	Periodontitis	0.321
	*Total*	0.994 ± 0.926 (32)	0.870 ± 0.175 (36)	0.989 ± 0.413 (38)	0.950 ± 0.572 (106)	COVID-19 x Periodontitis	0.258
**Average Creatinine** (mg/dL)	*MC*	0.918 ± 0.162 (23)	0.959 ± 0.178 (24)	1.071 ± 0.250 (11)	0.964 ± 0.192 (58)	COVID-19	0.068
	*SC*	1.565 ± 2.020 (9)	0.987 ± 0.153 (12)	1.230 ± 0.757 (27)	1.232 ± 1.026 (48)	Periodontitis	0.330
	*Total*	1.100 ± 1.076 (32)	0.968 ± 0.169 (36)	1.184 ± 0.652 (38)	1.085 ± 0.714 (106)	COVID-19 x Periodontitis	0.274
**Max Sodium** (mEq/dL)	*MC*	140.5 ± 3.0 (23)	141.6 ± 3.8 (24)	140.8 ± 2.9 (11)	141.0 ± 3.3 (58)	**COVID-19**	0.009 ^*^
	*SC*	138.3 ± 3.8 (9)	140.3 ± 2.8 (12)	139.2 ± 3.6 (27)	139.3 ± 3.5 (48)	Periodontitis	0.232
	*Total*	139.9 ± 3.3 (32)	141.2 ± 3.5 (36)	139.7 ± 3.5 (38)	140.3 ± 3.5 (106)	COVID-19 x Periodontitis	0.881
**Min Sodium** (mEq/dL)	*MC*	137.8 ± 2.7 (23)	137.2 ± 4.9 (24)	136.4 ± 2.8 (11)	137.3 ± 3.7 (58)	**COVID-19**	< 0.001 ^*^
	*SC*	133.2 ± 1.9 (9)	135.1 ± 4.1 (12)	134.1 ± 3.7 (27)	134.2 ± 3.6 (48)	Periodontitis	0.536
	*Total*	136.5 ± 3.2 (32)	136.5 ± 4.7 (36)	134.8 ± 3.6 (38)	135.9 ± 3.9 (106)	COVID-19 x Periodontitis	0.494
**Average Sodium** (mEq/dL)	*MC*	139.2 ± 2.1 (23)	139.5 ± 3.9 (24)	138.5 ± 2.0 (11)	139.2 ± 3.0 (58)	**COVID-19**	< 0.001 ^*^
	*SC*	136.0 ± 2.4 (9)	137.7 ± 2.1 (12)	136.8 ± 3.0 (27)	136.9 ± 2.7 (48)	Periodontitis	0.277
	*Total*	138.3 ± 2.6 (32)	138.9 ± 3.5 (36)	137.3 ± 2.9 (38)	138.1 ± 3.1 (106)	COVID-19 x Periodontitis	0.738
**Max Potassium** (mEq/dL)	*MC*	4.25 ± 0.47 (23)	4.34 ± 0.52 (24)	4.38 ± 0.32 (11)	4.31 ± 0.46 (58)	**COVID-19**	0.029 ^*^
	*SC*	4.64 ± 0.42 (9)	4.48 ± 0.36 (12)	4.49 ± 0.53 (27)	4.51 ± 0.47 (48)	Periodontitis	0.487
	*Total*	4.36 ± 0.48 (32)	4.39 ± 0.47 (36)	4.46 ± 0.48 (38)	4.40 ± 0.48 (106)	COVID-19 x Periodontitis	0.695
**Min Potassium** (mEq/dL)	*MC*	3.95 ± 0.50 (23)	3.75 ± 0.36 (24)	4.08 ± 0.46 (11)	3.89 ± 0.45 (58)	COVID-19	0.066
	*SC*	3.80 ± 0.32 (9)	3.73 ± 0.47 (12)	3.71 ± 0.46 (27)	3.73 ± 0.44 (48)	Periodontitis	0.454
	*Total*	3.91 ± 0.45 (32)	3.74 ± 0.39 (36)	3.82 ± 0.49 (38)	3.82 ± 0.45 (106)	COVID-19 x Periodontitis	0.422
**Average Potassium** (mEq/dL)	*MC*	4.10 ± 0.46 (23)	4.02 ± 0.39 (24)	4.22 ± 0.39 (11)	4.09 ± 0.42 (58)	COVID-19	0.653
	*SC*	4.22 ± 0.30 (9)	4.09 ± 0.30 (12)	4.11 ± 0.45 (27)	4.13 ± 0.39 (48)	Periodontitis	0.501
	*Total*	4.14 ± 0.42 (32)	4.05 ± 0.36 (36)	4.14 ± 0.43 (38)	4.11 ± 0.40 (106)	COVID-19 x Periodontitis	0.801
**Average Calcium** (mg/dL)	*MC*	8.50 ± 0.86 (9)	8.74 ± 0.84 (6)	7.90 ± 1.42 (4)	8.45 ± 0.98 (19)	Number of missing data exceeding analysis threshold	
	*SC*	8.80 ± 0.85 (2)	7.90 ± 0.67 (6)	7.77 ± 0.77 (14)	7.90 ± 0.77 (22)		
	*Total*	8.55 ± 0.83 (11)	8.32 ± 0.85 (12)	7.80 ± 0.90 (18)	8.15 ± 0.91 (41)		
**Average Phosphorous** (mg/dL)	*MC*	3.34 ± 0.74 (8)	3.09 ± 0.81 (6)	3.00 ± 1.01 (3)	3.19 ± 0.77 (17)	Number of missing data exceeding analysis threshold	
	*SC*	4.35 ± 1.91 (2)	3.76 ± 1.27 (5)	2.95 ± 1.12 (11)	3.33 ± 1.27 (18)		
	*Total*	3.54 ± 1.01 (10)	3.40 ± 1.05 (11)	2.96 ± 1.06 (14)	3.26 ± 1.04 (35)		
**Average Magnesium** (mg/dL)	*MC*	1.76 ± 0.12 (6)	1.98 ± 0.13 (5)	2.13 ± 0.32 (3)	1.92 ± 0.23 (14)	Number of missing data exceeding analysis threshold	
	*SC*	2.45 ± 0.64 (2)	1.88 ± 0.30 (4)	2.09 ± 0.70 (8)	2.08 ± 0.59 (14)		
	*Total*	1.93 ± 0.41 (8)	1.94 ± 0.22 (9)	2.10 ± 0.61 (11)	2.00 ± 0.45 (28)		
**Liver and Enzymes (patients excluded = 4)**							
**Max AST** (IU/L)	*MC*	60.7 ± 185.8 (26)	31.6 ± 19.6 (27)	39.5 ± 24.0 (11)	44.8 ± 118.9 (64)	COVID-19	0.120
	*SC*	101.3 ± 193.0 (10)	37.2 ± 36.5 (13)	274.8 ± 709.9 (30)	183.8 ± 547.0 (53)	Periodontitis	0.131
	*Total*	71.9 ± 186.0 (36)	33.4 ± 25.9 (40)	211.7 ± 613.7 (41)	107.7 ± 382.9 (117)	COVID-19 x Periodontitis	0.660
**Min AST** (IU/L)	*MC*	36.0 ± 65.3 (26)	24.5 ± 18.2 (27)	32.6 ± 21.2 (11)	30.6 ± 43.9 (64)	COVID-19	0.284
	*SC*	28.9 ± 14.3 (10)	26.7 ± 17.5 (13)	192.7 ± 681.6 (30)	121.1 ± 515.8 (53)	Periodontitis	0.172
	*Total*	34.0 ± 55.7 (36)	25.2 ± 17.8 (40)	149.8 ± 584.9 (41)	71.6 ± 349.8 (117)	COVID-19 x Periodontitis	0.829
**Average AST** (IU/L)	*MC*	50.1 ± 134.0 (26)	28.0 ± 17.8 (27)	36.1 ± 21.9 (11)	38.3 ± 86.2 (64)	COVID-19	0.204
	*SC*	55.1 ± 69.4 (10)	31.9 ± 23.1 (13)	226.8 ± 684.1 (30)	146.6 ± 520.2 (53)	Periodontitis	0.155
	*Total*	51.5 ± 118.6 (36)	29.2 ± 19.5 (40)	175.6 ± 588.8 (41)	87.4 ± 358.1 (117)	COVID-19 x Periodontitis	0.748
**Max ALT** (IU/L)	*MC*	53.3 ± 120.0 (26)	42.9 ± 33.9 (27)	43.1 ± 30.7 (11)	47.2 ± 79.8 (64)	COVID-19	0.125
	*SC*	108.3 ± 214.4 (10)	30.7 ± 21.8 (13)	211.8 ± 509.8 (30)	147.8 ± 398.9 (53)	Periodontitis	0.158
	*Total*	68.6 ± 150.8 (36)	38.9 ± 30.7 (40)	166.5 ± 440.9 (41)	92.8 ± 278.1 (117)	COVID-19 x Periodontitis	0.566
**Min ALT** (IU/L)	*MC*	43.2 ± 77.2 (26)	32.7 ± 28.9 (27)	32.0 ± 14.5 (11)	36.8 ± 52.6 (64)	COVID-19	0.218
	*SC*	58.9 ± 82.2 (10)	26.2 ± 14.9 (13)	155.1 ± 461.6 (30)	105.3 ± 351.3 (53)	Periodontitis	0.319
	*Total*	47.5 ± 77.7 (36)	30.5 ± 25.2 (40)	122.0 ± 396.9 (41)	67.8 ± 240.9 (117)	COVID-19 x Periodontitis	0.731
**Average ALT** (IU/L)	*MC*	48.6 ± 100.7 (26)	37.4 ± 29.6 (27)	36.6 ± 19.9 (11)	41.8 ± 66.9 (64)	COVID-19	0.171
	*SC*	77.8 ± 128.5 (10)	28.4 ± 17.9 (13)	173.7 ± 469.6 (30)	120.0 ± 360.6 (53)	Periodontitis	0.203
	*Total*	56.7 ± 108.0 (36)	34.5 ± 26.5 (40)	137.0 ± 404.7 (41)	77.2 ± 249.5 (117)	COVID-19 x Periodontitis	0.666
**Max Alkaline Phosphatase** (IU/L)	*MC*	200.6 ± 51.4 (26)	242.3 ± 116.1 (27)	216.9 ± 163.3 (11)	221.0 ± 105.9 (64)	COVID-19	0.255
	*SC*	260.0 ± 239.5 (10)	248.5 ± 141.7 (13)	254.9 ± 136.7 (30)	254.3 ± 158.1 (53)	Periodontitis	0.846
	*Total*	217.1 ± 131.8 (36)	244.3 ± 123.2 (40)	244.7 ± 143.2 (41)	236.1 ± 132.6 (117)	COVID-19 x Periodontitis	0.735
**Min Alkaline Phosphatase** (IU/L)	*MC*	196.4 ± 54.6 (26)	230.9 ± 122.5 (27)	205.4 ± 162.9 (11)	212.5 ± 108.9 (64)	COVID-19	0.455
	*SC*	239.2 ± 244.8 (10)	241.0 ± 147.8 (13)	219.0 ± 112.2 (30)	228.2 ± 150.2 (53)	Periodontitis	0.642
	*Total*	208.3 ± 133.8 (36)	234.2 ± 129.4 (40)	215.4 ± 125.7 (41)	219.6 ± 128.9 (117)	COVID-19 x Periodontitis	0.907
**Average Alkaline Phosphatase** (IU/L)	*MC*	198.6 ± 52.4 (26)	236.8 ± 118.5 (27)	211.1 ± 162.5 (11)	216.9 ± 106.8 (64)	COVID-19	0.342
	*SC*	250.1 ± 241.7 (10)	243.9 ± 145.3 (13)	237.0 ± 118.3 (30)	241.1 ± 151.0 (53)	Periodontitis	0.751
	*Total*	212.9 ± 132.4 (36)	239.1 ± 126.0 (40)	230.0 ± 129.9 (41)	227.9 ± 128.7 (117)	COVID-19 x Periodontitis	0.825
**Average Albumin** (g/dL)	*MC*	3.52 ± 0.54 (7)	3.60 ± 0.88 (6)	3.35 ± 0.35 (2)	3.53 ± 0.65 (15)	Number of missing data exceeding analysis threshold	
	*SC*	3.13 ± 0.33 (4)	3.21 ± 0.30 (7)	2.79 ± 0.58 (14)	2.96 ± 0.50 (25)		
	*Total*	3.38 ± 0.50 (11)	3.39 ± 0.64 (13)	2.86 ± 0.58 (16)	3.18 ± 0.62 (40)		
**Average Direct Bilirubin** (mg/dL)	*MC*	0.367 ± 0.306 (3)	0.285 ± 0.158 (8)	0.280 ± 0.179 (5)	0.299 ± 0.184 (16)	Number of missing data exceeding analysis threshold	
	*SC*	0.167 ± 0.058 (3)	0.680 ± 0.726 (3)	0.423 ± 0.323 (6)	0.423 ± 0.424 (13)		
	*Total*	0.267 ± 0.225 (6)	0.393 ± 0.396 (11)	0.358 ± 0.265 (11)	0.352 ± 0.310 (28)		
**Average Total Bilirubin** (mg/dL)	*MC*	0.717 ± 0.340 (3)	0.612 ± 0.256 (8)	0.660 ± 0.527 (5)	0.647 ± 0.349 (16)	Number of missing data exceeding analysis threshold	
	*SC*	0.467 ± 0.115 (3)	1.320 ± 1.316 (3)	1.102 ± 0.863 (6)	0.998 ± 0.876 (13)		
	*Total*	0.592 ± 0.265 (6)	0.592 ± 0.265 (11)	0.901 ± 0.733 (11)	0.797 ± 0.642 (28)		
**Markers**							
**Max LDH** (IU/L)	*MC*	593.8 ± 526.7 (26)	442.5 ± 209.1 (24)	442.8 ± 211.3 (11)	507.0 ± 381.4 (61)	**COVID-19**	0.003 ^*^
	*SC*	938.4 ± 785.0 (10)	701.4 ± 327.9 (13)	689.3 ± 475.2 (29)	740.2 ± 517.3 (52)	Periodontitis	0.239
	*Total*	689.5 ± 617.4 (36)	533.5 ± 281.9 (37)	621.5 ± 431.3 (40)	614.3 ± 462.0 (113)	COVID-19 x Periodontitis	0.826
**Min LDH** (IU/L)	*MC*	571.1 ± 537.4 (26)	401.1 ± 190.5 (24)	433.7 ± 209.3 (11)	479.4 ± 384.7 (61)	**COVID-19**	0.035 ^*^
	*SC*	695.1 ± 555.5 (10)	594.0 ± 215.8 (13)	629.2 ± 456.1 (29)	633.1 ± 425.2 (52)	Periodontitis	0.550
	*Total*	605.5 ± 537.4 (36)	468.9 ± 217.8 (37)	575.5 ± 410.3 (40)	550.1 ± 409.3 (113)	COVID-19 x Periodontitis	0.951
**Average LDH (IU/L)**	*MC*	583.6 ± 530.6 (26)	422.3 ± 196.2 (24)	438.3 ± 209.8 (11)	493.9 ± 381.5 (61)	**COVID-19**	0.010 ^*^
	*SC*	812.0 ± 636.8 (10)	639.6 ± 224.1 (13)	657.3 ± 461.8 (29)	682.6 ± 452.3 (52)	Periodontitis	0.350
	*Total*	647.1 ± 562.2 (36)	498.6 ± 228.9 (37)	597.1 ± 417.4 (40)	580.8 ± 424.3 (113)	COVID-19 x Periodontitis	0.985
**Max CK-MB (IU/L)**	*MC*	85.5 ± 162.9 (14)	25.4 ± 18.3 (15)	26.3 ± 7.6 (7)	48.9 ± 104.3 (36)	Number of missing data exceeding analysis threshold	
	*SC*	24.5 ± 17.7 (2)	24.4 ± 14.2 (10)	182.0 ± 730.1 (22)	126.4 ± 587.4 (34)		
	*Total*	77.8 ± 153.2 (16)	25.0 ± 16.5 (25)	144.4 ± 635.9 (29)	86.5 ± 414.8 (70)		

^*^Statistically significant

^a^All analyses adjusted for age, sex, and comorbidities

^b^“COVID-19” and “Periodontitis” are main effects; “COVID-19 × Periodontitis” is the interaction

#### Complete blood count

Periodontitis severity was associated with the minimum white blood cell (WBC) counts (*P* = 0.037). The interaction of periodontitis and COVID-19 was associated with the minimum WBC counts (*P* = 0.032); and inversely associated with the maximum, minimum, and average platelet counts (P values: 0.008, 0.001, and 0.002, respectively).

#### Venous blood gas

COVID-19 severity was associated with the average venous oxygen pressure (*P* = 0.022).

#### Coagulation

COVID-19 severity was associated with the maximum, minimum, and average prothrombin time (*P* values: 0.013, 0.016, and 0.010, respectively); the maximum, minimum, and average international normalized ratio (*P* values: 0.014, 0.026, and 0.014, respectively); and the maximum activated partial thromboplastin time (*P* = 0.016). Periodontitis severity was associated with the minimum prothrombin time (*P* = 0.047).

#### Kidney and electrolytes

COVID-19 severity was associated with the maximum and average urea levels (P values: < 0.001 and 0.003, respectively); the maximum creatinine levels (*P* = 0.042); and the maximum potassium levels (*P* = 0.029). COVID-19 severity was also inversely associated with the maximum, minimum, and average sodium levels (P values: 0.009, < 0.001, and < 0.001, respectively).

#### Liver and enzymes

Although there was a positive trend in liver enzyme levels with increases in the severity of COVID-19 and periodontitis, no statistically significant association was observed.

#### Markers

COVID-19 severity was associated with the maximum, minimum, and average lactate dehydrogenase levels (P values: 0.003, 0.035, and 0.010, respectively).

## Discussion

In this study, we investigated the association between the severity of COVID-19, the severity of periodontitis, and hematological tests in hospitalized patients. We found a significant association between the periodontitis severity and increased minimum WBC counts. The interaction between the periodontitis severity and COVID-19 severity was also found to be significantly associated with increased minimum WBC counts. There have been previous reports of increased WBC counts in the circulating blood of periodontitis patients in previous articles and meta-analyses [10, 30]. A similar finding was reported in the study done by Marouf et al. on the association between COVID-19 and periodontitis; they reported increased WBC counts in the more severe forms of periodontitis [17]. This increase in WBC counts can be attributed to the inflammatory response to periodontal disease, which causes an increase in WBC infiltration into periodontal tissues. The stimulation of bone marrow by local irritants and inflammation markers to produce more WBC, combined with the return of infiltrating WBC to the circulating blood, results in increased WBC counts [10]. We also found the interaction of periodontitis and COVID-19 to be associated with decreased platelet counts. None of the similar studies done on the association between periodontitis and COVID-19 reported any results regarding platelet counts [16–18]. Despite the fact that Botelho et al.'s meta-analysis on the hematological changes associated with periodontitis yielded no conclusive results regarding platelet count changes [10], they mentioned a positive trend towards increased platelet counts in aggressive forms of periodontitis and attributed it to stimulation of thrombocytogenesis due to increased hepatic thrombopoietin. On the other hand, the multiple meta-analyses done on the hematological changes associated with COVID-19 report decreased platelet counts, which are mainly attributed to coagulation changes and the hypercoagulable state associated with the disease pathogenesis. In our study, it appears that in milder forms of COVID-19, patients with severe periodontitis have increased platelet counts, while in patients with severe COVID-19, the platelet counts are affected by the more severe pathology of COVID-19 and are decreased.

We found COVID-19 severity to be directly associated with the average vSO2%; the maximum, minimum, and average PT; the maximum, minimum, and average INR; the maximum PTT; the maximum and average urea; the maximum creatinine; the maximum potassium; and the maximum, minimum, and average LDH. We also found COVID-19 severity to be inversely associated with the maximum, minimum, and average sodium levels. In the meta-analysis done by Ghahramani et al. [6] on the hematological changes associated with COVID-19, increases in urea, creatinine, LDH, and PT and decreases in platelet counts, albumin, and sodium were reported, which were similar to our findings. They also reported increased AST, ALT, bilirubin, ESR, CRP, procalcitonin, fibrinogen, and d-dimer, which did not reach statistical significance in our study. Soraya et al. reported increased WBC and CRP and decreased platelet counts in their meta-analysis on laboratory changes associated with COVID-19, which were in agreement with our findings. They also reported increased neutrophils and decreased lymphocytes [7]. We also found a similar trend but it did not reach statistical significance. Deng et al. [8] also reported similar findings in their meta-analysis on the COVID-19 associated hematological changes, including increased LDH and creatinine. They also reported increased AST, ALT, and CK-MB, and decreased albumin levels, which did not reach statistical significance in our study. We also found similar results to the meta-analysis done by Len et al. on the associated coagulation changes in COVID-19 patients, which included increased PT and PTT [9].

The aforementioned changes in hematological tests can be explained by many factors. The tests responsible for the function of organs, namely AST, ALT, urea, and creatinine, which are responsible for the assessment of the liver and kidney function, are affected by the COVID-19 associated organ involvement and the organ damages caused by the cytokine storm [23, 24, 31]. The increase in PT/INR and PTT and the decrease in platelet counts are explained by multiple phenomena. The COVID-19 infection causes an increase in coagulation tendency and platelet activation in the early stages. Following further progression of the disease, a hypercoagulable state develops, and the coagulation inhibition pathways are suppressed, which results in coagulation events such as DIC and disruptions in the coagulation tests [9, 31]. Additionally, anti-coagulation medications used to prevent vascular events in these patients are also partly responsible for the increases in the coagulation test results. LDH is a cytoplasmic enzyme present in most of the major organs. Increases in the levels of this enzyme are an indicator of the cellular damages caused by COVID-19, either directly caused by virus replication or indirectly caused by the cytokine storm [8, 31]. It has also been documented that levels of this enzyme can predict the survival of the patient [32].

Underlying mechanisms connecting periodontitis and COVID-19 are numerous, of which few have already been mentioned. The hyperinflammatory state, cytokine storm, and the organ damages caused by them is perhaps one of the prominent factors connecting both diseases and the hematological tests [23, 24, 31]. Perhaps the most prominent way in which periodontitis can affect COVID-19 is the elevation in systemic inflammation levels, with a multitude of cytokines reaching higher levels in more severe forms of periodontitis [29, 33–35], many of which are directly associated with COVID-19 progression and adverse outcomes [36–38]. The next connection can be the effect of periodontitis on systemic comorbidities [39], which would indirectly affect COVID-19 infection through the pronounced effects of these comorbidities on COVID-19 [40], many of which are already established [3, 41]. Last but not least, SARS-CoV-2 can independently replicate in the oral cavity and periodontium (as explained before, facilitated by entry receptors of these cells) and then spread through the body via hematologic, digestive, or respiratory routes [40], thus serving as a reservoir for COVID-19 infection. As more studies are conducted, more evidence regarding the underlying mechanisms connecting periodontitis and COVID-19 will emerge.

The main concerns about the analysis and comparison of the hematological tests’ results were the timings, the number of tests available for each patient, and the COVID-19 timepoint in which the test was taken. Patients with milder forms of COVID-19 were hospitalized for shorter durations and usually had fewer sets of tests with longer intervals between them. Although patients with severe/critical COVID-19 had more tests on average, the risk of a shift in the average results due to the severely affected tests at timepoints at which the patient’s condition was critical still remained a concern. We tried to alleviate this problem by analyzing the maximum, minimum, and average results (during the entire hospitalization course) of the tests whenever possible, which we think is a strength of our study. Another concern was the treatment medications. Even though many patients received similar principal medications, the dosages and responses might have been different between patients, some of which might affect hematological tests. We tried to rectify this problem by implementing stricter inclusion and exclusion criteria, eliminating patients with needs for medications that could potentially alter test results in significant ways. We also excluded patients with severe baseline organ deficiencies from the related hematological tests to further enhance the reliability of the results.

### Limitations and generalizability

One of the strengths of this study is that a periodontal diagnosis was made clinically, but at the same time, a limitation was the absence of radiographic data to further enhance the diagnosis. Another strength is that the study population consisted of hospitalized patients, which made studying more severe forms of COVID-19 in a more controlled environment possible. Another limitation was some missing data on some patients. In this study, we assessed the severity of COVID-19 retrospectively, which may be prone to inaccuracies or inconsistencies in the documentation. Although we tried to mitigate this issue by evaluating all available electronic and paper records during the entire hospitalization course, the issue still persists and is one of the limitations of this study. Another limitation was that the sampling day was not standardized between patients.

While studying hospitalized patients has provided us with the means to better evaluate more severe forms of COVID-19, a more reliable clinical appraisal of the patient’s condition, and a controlled environment to conduct hematological tests, it resulted in a limitation in that the findings of this study may not be reliably extended to the general COVID-19 population. The inclusion and exclusion criteria allowed us to better isolate the interaction between periodontitis and COVID-19 and to omit unaddressed potential confounding effects, but they may also limit the ability to generalize and extend the results to the general population. While we considered some confounders, such as age, sex, smoking, medications, and comorbidities, other confounders, such as socioeconomic status, BMI, and lifestyle, could not be accounted for, which may limit the validity of the results. To further investigate the issues addressed in this article, more studies with possible standardizations on test timings, and studies in which tests results are linked to disease course, possibly with prospective longitudinal or controlled trial designs are required.

## Conclusions

We found that the severity of periodontitis and COVID-19 are associated with changes in hematological tests, some of which are affected by the interaction between both diseases. The most prominent associations were with WBC, platelets, PT, PTT, urea, creatinine, potassium, sodium, and LDH.

It is well documented that periodontitis affects systemic health and systemic inflammation levels, and this study portrays periodontitis as a possible factor associated with more severe forms of COVID-19 and alterations in hematological tests. Based on these findings, it may be plausible to integrate oral hygiene measures with or without professional debridement into the treatment plan for COVID-19 patients to reduce the odds for COVID-19 progression and morbidities. Future research may reveal the exact underlying mechanisms that connect periodontitis to COVID-19.

## Data Availability

The data that support the findings of this study are available from the corresponding author upon reasonable request. The data are not publicly available due to legal restrictions.

## Abbreviations

ACE2: Angiotensin Converting Enzyme 2
ALT: Alanine Transaminase
AST: Aspartate Transaminase
CKMB: Creatin Kinase MB
CRP: C-Reactive Protein
ESR: Erythrocyte Sedimentation Rate
HP: Healthy Periodontium Group
ICU: Intensive Care Unit
IL-6: Interleukin-6
INR: International Normalized Ratio
LDH: Lactate Dehydrogenase
MC: Mild/Moderate COVID-19
MP: Mild/Moderate Periodontitis
NUL: Normal Upper Limit
PT: Prothrombin Time
PTT: Partial Thromboplastin Time
SC: Severe COVID-19
SP: Severe Periodontitis
vSO_2_: Venous Oxygen Saturation
WBC: White Blood Cell
